# Ten simple rules for aspiring graduate students

**DOI:** 10.1371/journal.pcbi.1009276

**Published:** 2021-08-19

**Authors:** Andrea I. Luppi, Charlotte Coco Newton, Lynde Folsom, Elisa Galliano, Rafael Romero-Garcia

**Affiliations:** 1 Division of Anaesthesia, School of Clinical Medicine, University of Cambridge, Cambridge, United Kingdom; 2 Department of Clinical Neurosciences, University of Cambridge, Cambridge, United Kingdom; 3 Department of Psychology and Centre for Brain Science, Harvard University, Cambridge, Massachusetts, United States of America; 4 Department of Physiology, Development and Neuroscience, University of Cambridge, Cambridge, United Kingdom; 5 Department of Psychiatry, University of Cambridge, Cambridge, United Kingdom; Carnegie Mellon University, UNITED STATES

## Introduction

Several supervillains have higher degrees—why don’t you? There can be a variety of reasons for wanting to go to grad school and for applying to a particular school and program. But often, one can only tell apart good and bad reasons from hindsight. Failing at something is perhaps the best way to know what can go wrong and what advice would have been useful when considering graduate school applications. We should know one of us started grad school 4 separate times, and another learned what a PhD was only after having started one. One of us lost 2 supervisors before even starting to write her thesis, and yet another accepted a PhD offer from the lab where she was working, without considering any alternatives; finally, one of us had applied to graduate schools for 5 years (with 19 rejections) before finally landing a PhD offer from their dream school. We hope that our hard-earned lessons will help you to avoid some of the pitfalls that we ourselves fell prey to. In this article, we address how to choose a graduate program, how to apply strategically, and some of the key challenges that may arise along the way toward graduate school. Conveniently, our advice can be summarized as 10 simple rules … so here they are.

Wait, you might think, there is already a *PLOS Computational Biology* article entitled “Ten Simple Rules for Graduate Students” [1]. Indeed, to foreshadow the Conclusions of this article, you should read that article! However, there are a number of important challenges that are specific to aspiring grad students: Should you attend graduate school in the first place? If so, what degree should you pursue? How to choose the right institution—or even the right country? Nowadays, an increasing number of countries across the world have established competitive graduate programs with English as the official language, aiming to attract international talent: Such a dazzling variety can be seemingly overwhelming. And, even once these choices are made, how does an aspiring graduate student become an admitted graduate student?

In other words, the excellent advice in “Ten Simple Rules for Graduate Students” [1] is primarily about being a grad student—whereas the article you are now reading is intended to help you in figuring out whether, where, and how to become a graduate student in the first place. So this is not a reboot of the 2007 article; it is a prequel.

### Rule 1: Choose who you want to be

Not all grad students are pursuing a doctorate, and not all those who are, are working toward a PhD (Box 1). As an article in *PLOS Computational Biology*, we expect that many readers may be considering a PhD or master’s degree in the sciences—but medical school, law school, and other advanced degrees can be substantially different from a PhD (just as a science PhD may be substantially different from a humanities PhD). Although we hope that much the advice in this article will be broadly applicable, we note that it is primarily written from the perspective of biological science PhD and master’s degrees.

So the first rule for aspiring grad students is to know what kind of grad student you are aspiring to be. And in reality, nobody aspires to be “a graduate student.” An undergraduate degree is education—part of your development as a well-rounded individual, and, therefore, arguably worth pursuing for its own sake. Graduate school, we would argue, is professional training: a means to an end. Those who attend medical school or law school don’t do so to be there: They do so to one day practice medicine or law. A master’s degree is a way to expand one’s skills and credentials in preparation for future study or employment, whether by deepening expertise in a familiar area or branching out to a new field. And a PhD is—in its broadest sense—a training toward independent thought and research. Although a PhD is a prerequisite for a career as a professor or principal investigator (PI), academia is by far not the only option for PhD holders—or even the most likely (see other articles in the Ten Simple Rules series on this topic). Therefore, consider carefully what is your end goal, as that is the fundamental determinant of what kind of graduate degree you should pursue—if any at all. Above all, be honest with yourself.

### Rule 2: Identify gaps to fill

If graduate school is a means to an end, then the right program (and degree type) for you is the one that best bridges the gap between who you are and who you want to be. This is, fundamentally, your reason for attending graduate school: You need to fill some gap in knowledge, skill, or qualification (broadly understood). So, perform a “gap analysis” [2]: Identify the gaps in your training, skills, knowledge, and credentials that you need to fill—letting this guide your choice of graduate program and where to pursue it. For example, a master’s degree can be focused on coursework and/or research, and a conversion course to change fields will be different from a specialization course in your field of interest—choosing between these options will benefit from an understanding of what gaps in your training you need to fill. Note that this does not need to be extremely specific: “I want to learn to do innovative research,” “I want to investigate cancer biology,” or “I want to acquire the qualification X that will allow me to apply to my dream job Y” may suffice as reasons for pursuing a graduate degree. This being said, the more specific your understanding of what you want to get out of graduate school, the more you will be able to narrow down on the right program.

In particular, PhD programs can vary substantially not just in their length (see below) but also in what they offer: Some require a choice of supervisor from the outset; others involve “rotations” in different labs (short periods of research intended to familiarize a student with a lab’s techniques, scientific questions, and environment, and help in the choice of supervisor). Some will involve coursework, others won’t. Working as a teaching assistant is typically a requirement in some countries (e.g., the US), but not in others. Be sure to look beyond a program’s label: For interdisciplinary fields such as neuroscience, programs and departments don’t always have “neuroscience” in the title, but neuroscientists can be found in departments ranging from medicine to psychology, engineering, and beyond; indeed, many institutions have interdepartmental programs for fields such as neuroscience, systems biology, or biomedical sciences. Be sure you understand what you need, and find a program that fills those gaps—ideally without filling your time with additional requirements you don’t need. These obligations will consume much of your time, so take seriously how you would like your time spent while in grad school.

If going for a PhD, you will also need to be able to identify another kind of gap: gaps in your field that you can fill with your research. Doctoral programs and institutions differ in when (and whether) they require their students to come up with a research proposal: In many cases, this is right at the point of application. In other cases, it is after completion of various qualifying exams. In yet other cases, the PhD funding is tied to a specific project set by the PI from the start. Of course, part of PhD training is about developing the required skills to find such gaps and learn to fill them: This is a valuable set of skills, and, indeed, acquiring them can be a worthy reason for embarking on a PhD program. Nevertheless, we would argue that it is helpful to have a burning “big picture” question that you want answered, and, ideally, a specific hypothesis that drives your learning and gives it direction [1]—while being aware that research questions naturally evolve as the research itself takes place and as a function of the lab’s direction. Keep track of the questions that spark your curiosity (literally, jot them down in a journal!): among them may lie the topic of your dissertation.

### Rule 3: Find a mentor who can help you fill those gaps

The idea of a PhD is based on the model of apprenticeship: You train under a recognized master of the profession for many years to learn their ways. However, academic research is driven by curiosity and passion (or so we would hope), and even in the same department, each individual will differ in what they are enthusiastic about. So, when you apply to a PhD program, ensure that there is at least 1 faculty member (and ideally more) whose interests you share and whose ways you wish to learn: not just their research techniques, but also their broader approach to science, mentoring, and academic life [3–5]. Note that this advice does not just apply to PhD applicants, but also to those wishing to pursue a master’s degree that is fully or heavily research based.

In addition to the hands-on versus hands-off continuum in supervision style, consider also academic seniority: Although your supervisor’s standing will be important in helping you get a job after graduate school [6], senior academics often have more demands on their time and may have less time to mentor individual students (especially if they run large labs). In contrast, a junior PI with a smaller lab may have more time and more recent experience with hands-on research and is likely to have more at stake in your success—but they may be less well connected and less experienced at managing a lab. Proactively considering the trade-offs between these aspects, and matching them to your own preferences, will help to ensure that you get the mentor that’s right for you [1].

Pay attention to where graduate students are on a supervisor’s papers: Do they get first-author publications, or are they relegated to the acknowledgments? Where do they end up after grad school—and how does that match with where you would like to end up? Contacting former students is a great way to find out about the lab’s culture: You can think of this as getting (informal) references on the supervisor, just as they are getting references on you from former mentors (more on this below).

Some programs require you to apply to work with a specific supervisor from the outset, whereas in other cases, the choice of lab is made later (especially for programs with a component of rotations, which can involve faculty from different departments). Rotations can be invaluable to “try out” a lab’s culture and a supervisor’s management style, but do not blindly assume that in any given institution there will be someone that you will enjoy working with: rather, make sure of it from the moment of application. Be aware that academics can move institutions, retire, or fail to get tenure: So, if possible, ensure that there is more than 1 faculty member whose lab you would be excited to join.

Do not be afraid to email your prospective supervisors and pitch an idea to them: You never know if spare funding or open-ended positions are available. Write a polite and concise email, tailored to the individual recipient and demonstrating your engagement with their specific research. Generic or copy-pasted emails will get you nowhere, but academics will appreciate genuine interest in their work.

### Rule 4: Fit over fame: Do not just choose where you want to “have gone”

Whether this ought to be the case or not, it is hard to deny that holding a degree from a prestigious institution will contribute to your “star power” and subsequent job prospects [6,7]—whether in academia or elsewhere. However, choosing a less suitable program just because of the school’s name will likely do you a disservice. Passion and the right environment breed excellence [1,8], and excellence shines on any CV, no matter where it was achieved.

A good fit (broadly defined—more on this below) will be key to keep you motivated throughout your graduate studies, because each step will feel like a step in the right direction: The direction of the goal that led you to attend graduate school in the first place. In contrast, a poor fit will sap your energy and motivation, potentially leading to burnout [9,10]. This is especially true for doctoral degrees, which typically take many years of intense and sustained effort (Box 1). Of course, no program is ever perfect, and you will have to “make the best of the courses you have at hand” [11] to some extent. However, if there is a systematic misalignment between your program and your personal goals and interests, you run the risk of losing your enthusiasm even for the things that brought you to grad school in the first place. So, make sure that your chosen program sparks your enthusiasm and that the department, lab, and mentor will share and nourish this enthusiasm.

**Box 1. Grad school ≠ doctorate ≠ PhD.**

- **Graduate (grad) school:** Any institution offering postgraduate degrees (i.e., requiring an undergraduate degree), such as doctorates and master’s degrees. Note that in the United States, law school and medical school are graduate schools, whereas in other countries, they are not, since medicine and law courses begin as undergraduate degrees.
- **Doctorate:** Any postgraduate degree conferring the title of “doctor.” This includes the PhD (“Philosophiae Doctor,” Latin for “doctor of philosophy”), but also the MD (doctor of medicine, typically granted by a medical school) and the JD (“Juris Doctor,” doctor of law, typically granted by a law school), among others.
- **PhD:** A postgraduate doctoral degree (sometimes instead abbreviated as DPhil) involving the completion of a substantial body of research in a specific academic discipline, conducted during several years of training under the supervision of an expert in the field. A PhD is a near-universal prerequisite for a career as a professor or PI.

So, a “graduate student” may be studying for a PhD or another type of doctorate, or, more broadly, any other kind of postgraduate degree, e.g., a master’s degree.

Of course, we acknowledge that it is not feasible to thoroughly research every faculty member from every program at every possible institution. Knowing what kind of program would suit your training needs (Rule 2) can be a good way to quickly narrow down your options (see also Rule 5 below about the importance of location). Likewise, if you already know what topic you want to pursue for your research, this can help to focus your search. Nevertheless, trade-offs between the depth and breadth of your search will inevitably emerge. While there is no one-size-fits-all approach, we would argue that the importance of fit means that it is advisable to make fewer but more carefully crafted applications (see also Rule 7 below).

### Rule 5: Choose a place where you can see yourself living

This is the other side of the “fit” question in Rule 4: You go to grad school to experience both professional and personal growth, so choosing a place where you can see yourself (and your family, if you have one) living and thriving for a number of years is key. Does the institution foster an inclusive community while respecting and welcoming diversity? More simply, will you feel safe and supported enough to be your fully creative and engaged self?

More broadly, consider also the geographical aspects: climate, culture, language, recreational and sport opportunities, proximity to nature or to big cities, and distance from home: Will you have access to what you need to de-stress and replenish your mental resources [5]? What kind of healthcare will be available, and will you be able to afford it? These are just some of the considerations that should factor into your decision, as well as job opportunities for your partner, availability of childcare, and the opportunity to settle permanently, if applicable to your situation. Likewise, law and medicine qualifications differ by countries, so the place where you attend graduate school may have a large impact on where you will be able to practice your profession. In summary, do not overlook the placement of the program in the world, beyond academia.

### Rule 6: Time is money—Invest both wisely

Although scholarships and other sources of funding can be available to cover the living expenses of a single student (Box 2), grad students are not known for living in luxury. So treat the decision to attend graduate school for what it is: a multiyear financial investment, for which you need to plan accordingly. Consider carefully (and well in advance!) the available funding options—both within and outside of the specific institution (e.g., government scholarships; Box 2).

**Box 2. Funding your graduate studies.**

Graduate students typically need funding to cover (i) tuition fees; and (ii) living expenses (which may include health insurance and other costs). Although self-funding (e.g., through loans) can be an option, funding for a PhD or master’s degree can also come from different sources.

- **Scholarship/fellowship/award:** Although the name can vary by country and institution, this is when some entity pays for your tuition fees and/or living expenses. Many countries have some funding of this kind available, and so do many universities—often on a competitive basis and with specific eligibility criteria. Charitable foundations are another potential source of this kind of funding.
- **Studentship/pre-funded project:** A department or individual researcher may have secured funding for a PhD student to work on a specific research project, for example, as part of a grant they obtained.
- **Research/teaching assistantship:** Tuition and/or stipend are provided in exchange for services rendered to the department, such as assisting with teaching duties, or working as a laboratory technician/assistant alongside one’s PhD work.

Note that legal status of your funding (e.g., taxable versus tax free) can vary by country; likewise, in some countries, graduate students who perform research or contribute to teaching activities are classed as employees, whereas in other countries, they are not. Finally, some countries allow international students to work outside of campus while enrolled on a graduate course, whereas others do not or have limits.

It is also important to be aware that the cost of a graduate degree can vary enormously by country, institution, and degree type: Some countries have minimal or even no fees (e.g., tuition fees in several West European ones countries are less than $1,000 USD per year); others can have price tags of several tens of thousands of USD. Note that despite their shorter duration, the cost for master’s degrees can exceed that of PhDs, sometimes by a substantial margin.

In particular, PhDs can vary considerably in duration, with US PhDs taking sometimes twice as long as the same qualification in other countries (e.g., 6 to 7 years, or even longer in the humanities, compared with 3 to 4 years in many other countries). If what you want from a PhD is learning to carry out independent research, and credentials that demonstrate it, then a shorter PhD should suffice to achieve this. A longer PhD will give you more time to carry out long-term projects and accumulate publications, which may be desirable if a career in academia is your end goal—although this can be made up through longer postdoctoral training. The length of your permanence in graduate school(s) will have important financial implications: As the old adage goes, “time is money” [11]. So be aware of the differences, then choose what most suits your needs and aspirations.

Consider also whether you need a master’s degree first. In many countries, higher education is organized in a 3 tiers system: Undergraduate studies are followed by a master’s degree (often 2-year long), and only then a student becomes eligible to apply for a PhD. However, this is not always the case: In the United Kingdom, for instance, it is often possible to skip the second step and obtain a PhD within 3 years of finishing one’s undergraduate degree. Moreover, several countries offer integrated master’s-plus-PhD programs. Besides fulfilling eligibility requirements, one needs to consider that a master’s degree (or working as a lab manager/research assistant) can be a great way to try out life in a new place (see Rule 5 for the importance of this) and assess whether you truly enjoy the day-to-day aspects of life as a grad student. However, beware of using the master’s route as an excuse to simply put off committing to a PhD: Being honest with yourself is your best policy [6]. On the other hand, a master’s degree can represent a valuable investment also for those who have no intention of pursuing a PhD afterwards, in terms of acquiring desirable skill and qualifications—but beware that a shorter duration may not always translate to lower cost (Box 2).

### Rule 7: Your admission should be a win for everyone

Graduate school admissions are not just an investment on your part: When a program accepts a graduate student, the department and the broader institution are committing to many years’ worth of support for your development and research. More concretely, faculty members are committing to have you as their junior colleague for many years. Mentor–mentee relationships often last even beyond the end of graduate school, sometimes spanning decades. Students can have a substantial impact on a lab’s research direction, but also on the broader departmental and campus culture (from open science practices to public engagement), in virtue of their personality, contribution to teaching, and involvement in departmental or student committees.

So, it is wise to demonstrate your appreciation for these subtler but crucial aspects of what goes into an admissions decision. When writing your statement of motivation (see Box 3 and Rule 8), don’t only tell the committee why that program is right for you. Instead, be sure to also demonstrate why they should want you to join their department and community. Are you collaborative and helpful, the kind of colleague who can support and inspire those around them? Cohort composition can be an important factor in determining who will be admitted: You need to convince the admissions committee that you will contribute to the success and thriving of other people in your cohort and your lab and to the broader community of the department. In other words, convince them that they want to have you around, not just that you want to be around them!

**Box 3. Components of a typical graduate application package.**

The requirements for application can vary by country and institution, and we recommend that you check them well in advance. For instance, some institutions may require standardized testing such as the Graduate Record Exam [12], and you will need to arrange for those to be completed before the application deadline. Here, we focus on some aspects that tend to be common across many countries, institutions, and degrees.

- **Statement of purpose/motivation statement/letter of intent:** This is a document, typically in essay style, where the applicant conveys their suitability for the program and institution, drawing on their own personal interests and trajectory.
- **Research proposal:** Typically required for PhDs that involve directly joining a lab to work on a specific project defined by the applicant. This document outlines the rationale for the project and how the applicant intends to address it. Typically, when a research proposal is required, the applicant also needs to have identified a relevant supervisor to develop the research proposal together (as a necessary but not sufficient condition for admission).
- **Curriculum vitae/resume:** A comprehensive but succinctly written list of the applicant’s education and employment history (classes taken and grades) and achievements—often accompanied by official degree transcripts.
- **Recommendation letters/letters of support:** Letters from former employers or academic advisors detailing why (and whether) the applicant is suitable for the position and how they rank in relation to their peer cohort.
- **Interview:** Shortlisted candidates may be invited (in person or online) to 1 or more interviews, which may also include a campus visit or other orientation activities. Interviews may be with a panel or a single faculty member and may involve a presentation by the candidate (e.g., of a project that they have been involved with in the past, of the research project that they proposed for the PhD, or a paper that the interview panel has selected).
- **Bureaucracy:** International applicants often face additional hurdles that can be costly and time consuming. Although this will be country specific, 2 common requirements include visas and language tests (if you are not a native speaker of English and/or the local language). We emphasize the need to consider these aspects well in advance to give yourself the time to prepare and meet the requirements.

### Rule 8: Show, don’t tell: Build synergy between CV and motivation statement

Your statement of purpose or motivation is a major factor to getting an interview for grad school and your first real opportunity to show who you are and what you want. Being an academic is a multifaceted profession that encompasses not only research but also teaching, leadership, teamwork, and communication. What unique experiences, skills, and perspectives do you bring to the role? Get into the mindset that you are the asset they want, and demonstrate it with specific examples. Your statement is also an excellent way to show that you are serious about the department’s investment in you, by showing that you have already invested time and effort in them. So, don’t just copy web page slogans, spend time to research the program and department and how you fit within it thoroughly, and let this be reflected in a targeted, well-polished statement (Rules 7–9 from “Ten Simple Rules for Writing a Postdoctoral Fellowship” will come to your aid here [13]). Tell your own story, not what you think the assessors want to read.

A near-universal principle of successful statements is “show, don’t tell”—especially if you are writing for a panel of academics, who are trained to abhor unsubstantiated claims. Anyone can type the words “I am hardworking and motivated,” but not everyone can provide evidence of hard-won achievements, adversities overcome, or lessons learned from failure and having the grit to get back up [14]. So let the facts speak for themselves.

Beware, though: Your statement should not be redundant with your CV, which the admissions committee will also have. Instead, aim to build synergy between statement and CV. The statement is your own description of your academic arc and of how it leads to grad school as the logical next step. It’s your chance to guide the committee’s interpretation of your CV and provide context: What are the most salient elements, and what do they say about how you meet the criteria for becoming a successful graduate student? How competitive was that prize you won, and what qualities does it reflect? How did you manage to do well in your classes while also being a high-performing athlete? You can think of your CV as the Results section of a paper, providing the facts and evidence, and your statement as the Discussion [15]. An evidence-based statement will also help you to steer clear of both arrogance and false modesty, neither of which will serve you well.

Finally, identify carefully who could write a good letter of recommendation for you. Good letters will build on your specific personal arc and add evidence of your abilities and suitability as a graduate student, so choose writers who (i) know you well; and (ii) can speak from a position of authority about your strengths as a future academic/lawyer/clinician. Be sure to send them your CV and motivation statement (with plenty of time!), so that they can write targeted recommendations that enhance the synergy of your entire application package.

### Rule 9: Make the most of your interviews

Based on our experience with graduate school interviews across many institutions and countries, we suggest that the criterion for determining whether an interview went well should not be whether you get an offer in the end, but rather whether you had a good conversation. The faculty may seek to push you, to see how you respond to an intellectual challenge—not unlike a departmental seminar. Of course, you are applying to become a student, so it is okay to admit that you don’t know something—it’s a chance to demonstrate your genuine desire to find out the answer. However, being genuine does not mean being unprepared: Practice mock interviews, and if possible, find out who will be interviewing you, and become familiar with their interests and perspectives to make sure you can have an engaging conversation. And remember: You’re interviewing them too, as you are also trying to assess if this is the program that you want to attend. So don’t lose sight of this objective.

Likewise, this may be your chance to meet other applicants and current graduate students: Your peers who may become future friends and collaborators will be an important determinant of your experience in graduate school. Above all, remember: An interview is a chance to spend time with the undivided attention of some leading experts in your subject, talking about your field of interest. So try to enjoy this opportunity!

### Rule 10: Learn to fail, and learn from failure

Realize that grad school applications are themselves part of learning how to be an academic. A PhD application is a request for an institution to invest time and money in you, so that you can pursue a topic you’re passionate about. At its core, this is the cornerstone of an academic’s life: a grant proposal (even more so in cases where a specific research proposal is required as part of the application).

Like with grant applications, you are competing for a limited number of resources with many well-qualified people, and many of them will be just as talented and motivated as you (and they may even have read this very article!). Like grantmakers, admissions committees have to make very difficult decisions: Departmental politics come into play, as does the overall composition of the cohort. In the end, admissions committees consist of human beings, who can be tired or hungry and even—dare we say it—make mistakes. Sometimes, with graduate applications, failure really isn’t about you.

Failure is the bread and butter of a researcher’s life [16]. So be prepared for failure, and plan accordingly: Play the numbers game (but make sure you only apply for places that you genuinely want to take!), and set up backup options that you can fall back to while you prepare for the next round of applications. Beyond the sting of rejection, there may be a lesson to be learned and an occasion to improve. Solicit feedback, then revise and recycle your application [13,15]: Remember, all of your next applications will be better as a result of each single rejection!

Knowing how to turn failure into growth is itself a skill that needs to be cultivated, and it will be invaluable in any career path. So learn to fail well: Find a way to learn from your mistakes, to make better mistakes tomorrow. And remember that in the end, you only need 1 successful application—even if it comes after 19 rejections.

## Conclusions

We hope the rules outlined here will help you decide whether to go to graduate school, and, if so, where and how to apply. If you get that coveted letter that says “Congratulations”—go celebrate! But then, before you start, make sure you set aside some time to read “Ten Simple Rules for Graduate Students” [1] and “Ten Simple Rules for Finishing Your PhD” [17] and learn about the potential opportunities and pitfalls facing you in your next career step. Many articles in the “Ten simple rules” series are designed with that preparation in mind, so take advantage of these resources. There isn’t (yet) an article with “Ten simple rules for learning to fail”—but this article itself is the fruit of our failures, leading us to realize what we wish we had known when we were aspiring grad students. Following the 10 simple rules laid out here won’t protect you from failure, but it will allow you to avoid some of the classic failure modes—leaving you free to fail in more interesting and informative ways. Welcome to grad school!
